# Corrosion Behavior of 347H Stainless Steel in NaCl-KCl-MgCl_2_ Molten Salt: Vapor, Liquid, and Interface Comparison

**DOI:** 10.3390/ma18143412

**Published:** 2025-07-21

**Authors:** Zhiwen Liu, Huigai Li, Yang Wang, Yanjie Peng, Luyan Sun, Jianping Liang

**Affiliations:** 1 College of Materials Science and Engineering, Shanghai University, Shanghai 200072, China; liuzhiwen@sinap.ac.cn (Z.L.); lihuigai@shu.edu.cn (H.L.); 2 Shanghai Institute of Applied Physics, Chinese Academy of Sciences, Shanghai 201800, China; wangyang@sinap.ac.cn (Y.W.); pengyanjie@sinap.ac.cn (Y.P.); sunluyan@sinap.ac.cn (L.S.)

**Keywords:** 347H stainless steel, molten chloride salt, corrosion behavior, concentrated solar power

## Abstract

The suitability of 347H stainless steel (SS347H) for chloride salt environments is critical in selecting materials for next-generation concentrated solar power (CSP) systems. This study investigated the corrosion behavior of SS347H in a ton-scale purification system with continuously flowing chloride salt under three conditions: exposure to NaCl-KCl-MgCl_2_ molten salt vapor, immersion in molten salt, and at the molten salt surface interface. Results revealed that corrosion was most severe in the molten salt vapor, where HCl steam facilitated Cl^−^ reactions with Fe and Cr in the metal, causing dissolution and forming deep corrosion pits. At the interface, liquid Mg triggered displacement reactions with Fe^2+^/Cr^2+^ ions in the salt, depositing Fe and Cr onto the surface, which reduced corrosion intensity. Within the molten salt, Mg’s purification effect minimized impurity-induced corrosion, resulting in the least damage. In all cases, the primary corrosion mechanism involves the dissolution of Fe and Cr, with the formation of minor MgO. These insights provide valuable guidance for applying 347H stainless steel in chloride salt environments.

## 1. Introduction

Next-generation concentrated solar power (CSP) technology has gained increasing attention for its high operating temperatures, improved power generation efficiency, and enhanced economic benefits [1,2,3,4]. Among potential thermal storage and heat transfer media, molten chloride salts (e.g., NaCl-KCl-MgCl_2_) are highly promising due to their excellent thermophysical properties, including low viscosity, high specific heat capacity, high thermal stability (>800 °C), and low cost (<0.35 USD∙kg^−1^) [5,6,7,8,9]. However, unlike commercially used molten nitrates, molten chlorides are more corrosive to metallic structural materials at elevated temperatures [10,11,12]. This poses a significant challenge for the selection of materials for CSP systems. The choice of materials is critical to ensure the reliability and efficiency of the system over the long term [13].

347H stainless steel (SS347H) is a strong candidate due to its excellent high-temperature mechanical properties and relatively good corrosion resistance [13,14,15]. It has already been used in hot-salt piping and storage tanks in second-generation CSP systems using nitrate-based salt [16,17]. However, its performance in the aggressive chloride salt environments of Gen3 CSP systems remains insufficiently understood.

Molten NaCl-KCl-MgCl_2_ salts exhibit high vapor pressures at elevated temperatures, and the decomposition of hydrated chlorides and impurities can release gaseous oxides that accelerate corrosion [18,19,20]. A key corrosion mechanism for stainless steels is the preferential dissolution of chromium (Cr), often leading to subsurface cavity formation [21,22,23]. While prior studies have largely focused on corrosion in either molten salt or its vapor phase, the interface region, where complex interactions occur under real working conditions, has been less explored.

This study aims to address this gap by systematically comparing the corrosion behavior of SS347H under three conditions: in the molten salt vapor, within the molten salt, and at the interface between them. Using industrial-scale salt production equipment, corrosion mechanisms were investigated under each condition. Results show that corrosion is most severe in the vapor region, followed by the interface, and least severe in the molten salt. These findings provide valuable insights for optimizing the application of SS347H in Gen3 CSP systems.

## 2. Materials and Methods

### 2.1. Materials

The material used in this study was SS347H, cut into sheet samples measuring 150 × 20 × 3 mm^3^. Prior to corrosion testing, the sample surfaces were mechanically ground, polished, and then ultrasonically cleaned. The chemical composition of SS347H is shown in Table 1.

The molten salt used was a ternary eutectic mixture of NaCl-KCl-MgCl_2_, consisting of 24.50 wt.% NaCl, 20.55 wt.% KCl, and 54.95 wt.% MgCl_2_.

### 2.2. Experimental Procedure

**Figure 1:**
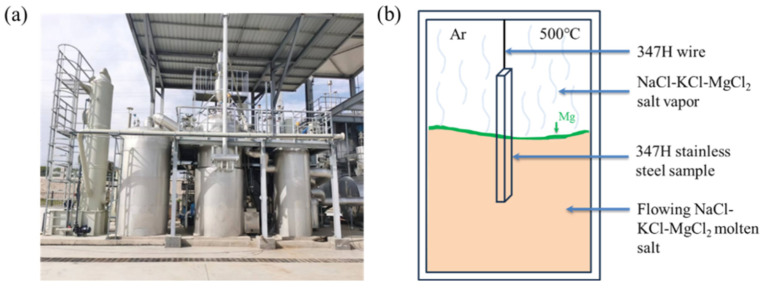
(**a**) Physical setup of the experimental apparatus. (**b**) Schematic of the corrosion experiment.

Corrosion experiments were conducted using a ton-scale continuous purification system for chloride salts (Figure 1a). This system enables continuous salt circulation and impurity removal (e.g., moisture and oxygen) via controlled drying and magnesium addition. Unlike static crucible setups, the flowing salt in this system interacts dynamically with internal piping, creating variable corrosion conditions.

To simulate real-world Gen3 CSP operating conditions, the sample was suspended in the molten salt tank using SS347H wire, with one end immersed in the salt and the other exposed to argon atmosphere above the salt surface (Figure 1b). This configuration created three distinct exposure regions: (1) molten salt region (fully immersed), (2) molten salt–vapor interface, and (3) molten salt vapor region (argon + salt vapor atmosphere).

The flowing molten salt caused slight surface agitation, intermittently exposing the interface region. Inhibitory elemental magnesium, added to purify the melt, floated at the interface due to its lower density.

The corrosion test was continued for 100 h at 500 °C. After exposure, the sample was cooled and ultrasonically cleaned sequentially with tap water, deionized water, and anhydrous ethanol, and then dried with cool air. After cleaning, thickness measurements were taken at five positions within each region, with three repeated readings per position to ensure data accuracy. Corrosion behavior was evaluated separately for the vapor, interface, and molten salt regions.

### 2.3. Characterization

Microstructural analysis of the pristine and corroded SS347H samples was performed using X-ray diffraction (Bruker D8 Advance, Billerica, MA, USA). Morphology and elemental analysis before and after corrosion was characterized using a scanning electron microscope (SEM, Zeiss Merlin FE, Oberkochen, Germany) coupled with an energy-dispersive spectrometer (Oxford Instruments X-Max 80 mm^2^, Abingdon, UK). All SEM analyses were performed at an accelerating voltage of 15 kV.

## 3. Results and Discussion

### 3.1. Macroscopic Morphology and Crystalline Structure

**Figure 2:**
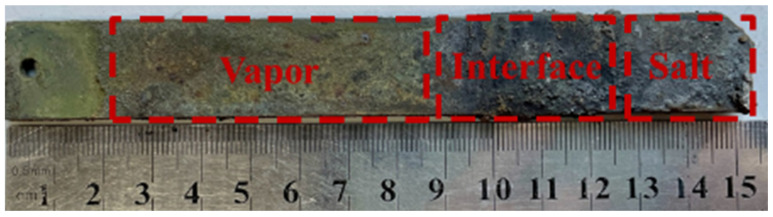
Macroscopic surface morphology of SS347H after corrosion in different regions.

Upon removal from the corrosion apparatus, the surface of SS347H showed distinct visual differences across three exposure regions (Figure 2). The molten salt vapor region appeared yellow-green in appearance, the interface region featured a thick, uneven, black deposit layer, and the molten salt region had gray deposits. Macroscopic observations indicated that the most severe corrosion product accumulation occurred at the interface region.

Sample thicknesses were measured separately in each region. After corrosion and subsequent cleaning, the measured thicknesses of the metals—including the effects of corrosion products—were 3.21 mm in the vapor region, 4.39 mm at the interface, and 3.32 mm in the salt region. Given the original thickness of 3 mm, corrosion products accumulated in all regions, with the highest accumulation at the interface.

**Figure 3:**
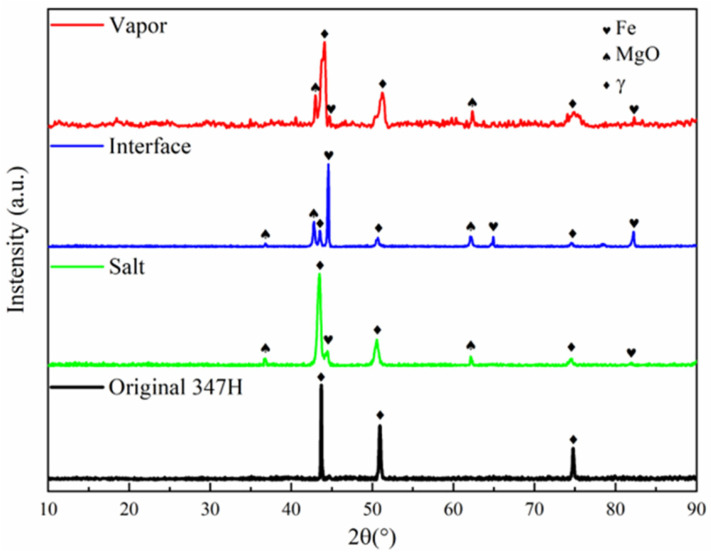
XRD patterns of different corrosion regions of SS347H after 100 h of exposure to NaCl-KCl-MgCl_2_ molten salt.

XRD analysis revealed distinct phase transformations in the three regions (Figure 3). The austenitic structure of SS347H remained predominantly stable across all regions, with additional diffraction peaks corresponding to Fe and MgO. Notably, the interface region exhibited strong Fe peaks surpassing those of the matrix, along with pronounced MgO peaks, indicating more complicated corrosion at this interface. The appearance of strong iron peaks will be discussed in Section 4.

### 3.2. Surface Morphology and Elemental Distribution

**Figure 4:**
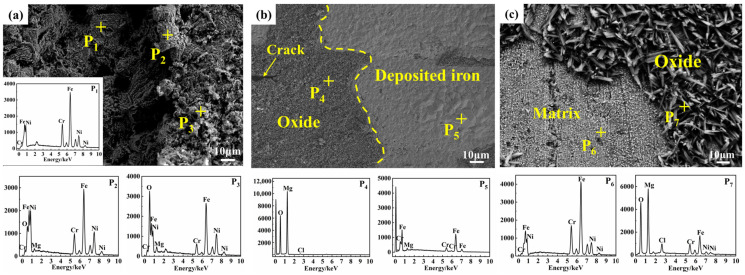
Surface morphology of SS347H after 100 h of corrosion in NaCl-KCl-MgCl_2_ molten salt: (**a**) vapor region, (**b**) interface region, and (**c**) molten salt region.

SEM images and EDS analysis (Figure 4) revealed region-specific surface characteristics and elemental distributions. In the vapor region, the surface appeared uneven, featuring irregular, deep corrosion pits (Figure 4a). EDS analysis revealed both exposed substrate (P_1_) and progressive oxidation of the substrate (P_2_, P_3_).

In the interface region, the surface was relatively flat and exhibited two distinct areas. On the left side of Figure 4b, a dense, dark gray oxide layer showed high O and Mg content (P_4_), indicating MgO formation. On the right side, a smooth, light gray area was primarily composed of Fe and Cr.

In the molten salt region, two distinct areas were also observed. Some areas showed exposed metal substrate, with Cr, Ni, Mg, and O detected by EDS (P_6_). Other areas showed rough, feather-like structures, with MgO identified as the dominant component (P_7_).

### 3.3. Cross-Sectional Morphology and Element Distribution

**Figure 5:**
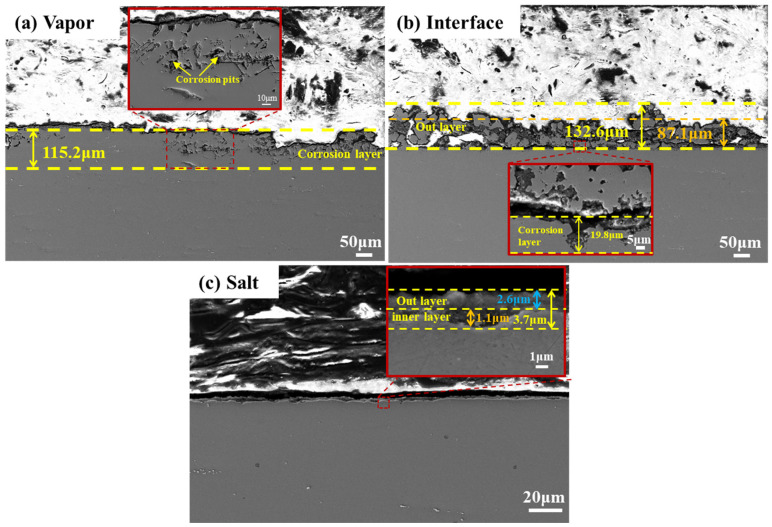
Cross-sectional morphology of SS347H after 100 h of NaCl-KCl-MgCl_2_ molten salt corrosion: (**a**) vapor region, (**b**) interface region, and (**c**) molten salt region.

Cross-sectional SEM analysis (Figure 5) further clarified corrosion differences. In the vapor region, corrosion was characterized by deep, uneven corrosion pits with maximum depth 115.2 μm. In the interface region, a double-layer structure was observed: an outer granular deposit layer (pure iron and MgO) with an average thickness of 87.1 μm and a maximum of about 132.6 μm, and an inner corrosion layer of 19.8 μm. The molten salt region showed a relatively flat surface with no significant corrosion pits or defects. The total corrosion depth was 3.7 μm, consisting of a 2.6 μm outer layer and a 1.1 μm inner corrosion layer. Based on the maximum corrosion depth, the corrosion rates of SS347H after 100 h of exposure were 1.2 µm/h in the molten salt vapor region, 0.2 µm/h at the interface, and 0.04 µm/h in the salt region. The estimated corrosion rates may be higher than in real-world applications due to accelerated test conditions and the use of maximum depth. However, the aim of this study is to compare corrosion severity across the three regions, so the relative values are more meaningful than the absolute rates.

These results indicate that corrosion severity followed the order vapor region > interface region > salt region. This trend contrasts with the macroscopic observations discussed in Section 3.1, where the interface region appeared to exhibit the most severe corrosion. The prominent visual degradation at the interface is attributed to the accumulation of thick and uneven corrosion product layers—primarily Fe and MgO—formed through displacement reactions between Mg in the molten salt and Fe^2+^/Cr^2+^ ions. These reactions deposit metallic Fe and Cr on the surface and promote MgO formation, leading to substantial surface buildup without significant penetration into the substrate.

In contrast, cross-sectional SEM analysis provides a direct assessment of material degradation. It reveals that the vapor region experiences the deepest corrosion penetration (~115 µm), mainly due to the aggressive attack by volatile HCl gas on Fe and Cr. Despite its more severe surface deposits, the interface region shows a much shallower penetration depth (~20 µm), highlighting the difference between surface appearance and actual material loss.

**Figure 6:**
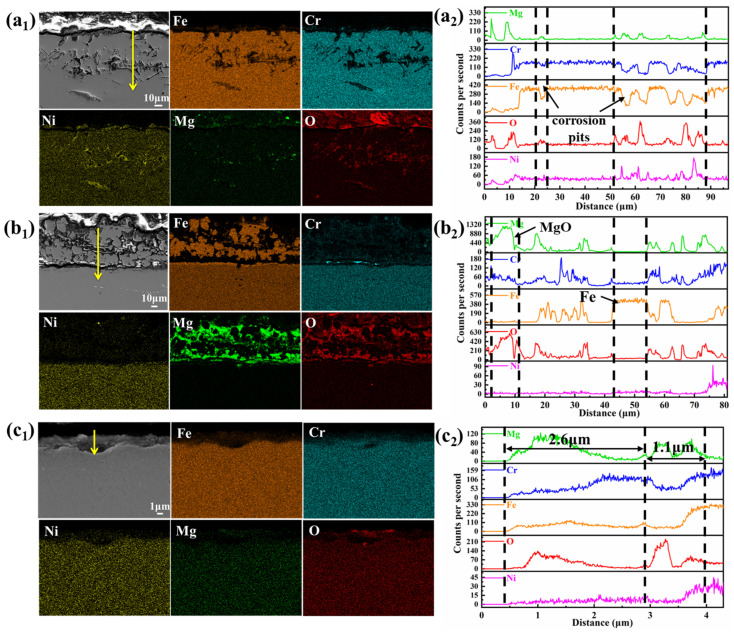
Element distribution and EDS results of SS347H after corrosion in three regions: (**a_1_**,**a_2_**) vapor, (**b_1_**,**b_2_**) interface, and (**c_1_**,**c_2_**) molten salt.

EDS elemental mappings (Figure 6) highlighted compositional changes. In the vapor region, corrosion primarily involved the dissolution of Cr and Fe, leading to their depletion, while Ni became enriched in the corrosion pits. The line scan results (Figure 6(a_2_)) confirm that Fe and Cr decreased in the pits, while Mg, O, and Ni concentrations increased. Mg and O infiltrated the corrosion region, forming MgO, with some oxide adhering to the surface. In the interface region, significant Fe deposition and MgO formation were observed (Figure 6(b_1_,b_2_)), along with some Cr enrichment, distinguishing it from the corrosion observed in the vapor region. The corrosion inhibitor Mg effectively reduced corrosion in the interface and salt regions but had a limited effect on vapor-phase corrosion. In the molten salt region, minimal corrosion was observed, with only a small amount of corrosion products in the outer layer and depletion of Cr and Fe in the inner layer. The outer corrosion layer had increased concentrations of Mg, O, and Cr, indicating the presence of MgCr_2_O_4_. The inner layer contained MgO, confirming the reaction of Mg and O in this region.

## 4. Corrosion Mechanism Discussion

**Figure 7:**
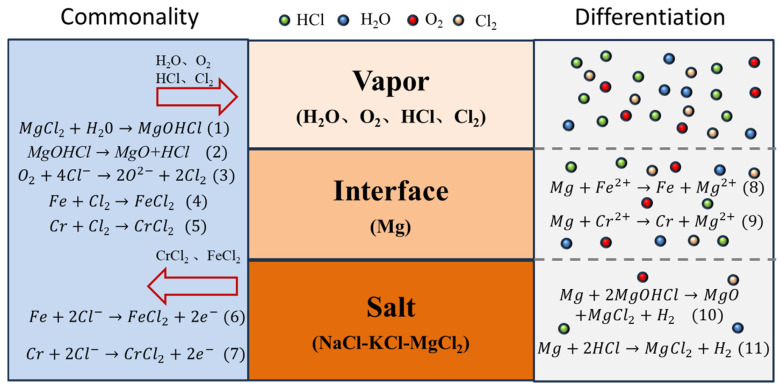
Schematic corrosion mechanism of SS347H in NaCl-KCl-MgCl_2_ molten salt.

The corrosion mechanism of SS347H in molten chloride salt is primarily governed by reactions between metallic elements (Fe and Cr) and corrosive species such as MgOHCl, which forms predominantly due to the hygroscopic nature of MgCl_2_. At elevated temperatures, MgOHCl decomposes into MgO and highly corrosive HCl, significantly accelerating the corrosion process. The corresponding chemical reactions are shown as Equations (1) and (2) in Figure 7.

The generated HCl volatilized into the vapor phase, interacting aggressively with metallic surfaces and exacerbating corrosion. This reaction led to the degradation of the steel and formation of MgO as corrosion products on the surface. Cross-sectional analyses (Figure 3, Figure 4 and Figure 6) confirmed significant Cr and Fe depletion in the vapor region, highlighting the severe impact of gaseous HCl.

In the interface region, magnesium-based inhibitors floated on the molten salt surface and preferentially reacted with corrosive compounds (e.g., H_2_O). This reaction enhanced molten salt purity and effectively reduced chromium dissolution, forming protective magnesium oxides. Additionally, magnesium underwent displacement reactions with Fe^2+^ and Cr^2+^, depositing metallic Fe and Cr layers, clearly visible in Figure 6(b_1_).

In the molten salt region, continuous removal of impurities by magnesium inhibitors minimized corrosive elements (H_2_O and O_2_), resulting in the highest salt purity and minimal corrosion.

These corrosion processes are summarized schematically in Figure 7. All three regions shared common corrosion pathways involving chemical reactions with H_2_O and O_2_, producing corrosive substances such as Cl_2_ and HCl, and causing dissolution of Fe and Cr into ionic forms. Variations in corrosion severity were due to differing concentrations of Cl^−^, H_2_O, and O^2−^. The highest concentrations in the vapor region resulted in the most severe corrosion, while the lowest concentrations in the molten salt region resulted in minimal corrosion. Magnesium-driven displacement reactions at the interface further altered the corrosion dynamics.

## 5. Conclusions

This study systematically investigated the corrosion behavior of SS347H in molten NaCl-KCl-MgCl_2_ salt under three exposure conditions: vapor, interface, and molten salt regions. The results demonstrated that corrosion severity followed the order vapor > interface > salt. The molten salt vapor region experienced the deepest corrosion due to high concentrations of volatile HCl and aggressive oxidative species, leading to substantial Cr and Fe depletion. The interface region showed notable corrosion product accumulation and deposition of Fe and Cr via magnesium-induced displacement reactions. In contrast, the molten salt region exhibited the least corrosion, benefiting from continuous impurity scavenging by magnesium, which effectively inhibited the corrosion process.

These findings highlight the importance of impurity control and corrosion inhibitors in chloride salt environments and provide valuable guidance for the safe and durable application of SS347H in next-generation concentrated solar power systems.

## Figures and Tables

**Table 1:** Chemical composition of SS347H (wt.%).

Material	C	Si	Mn	Cr	Ni	Nb	Ti	N	Fe
Composition	0.05	0.26	1.58	18.06	9.40	0.47	0.005	0.010	Bal.

## Data Availability

The raw data supporting the conclusions of this article will be made available by the authors on request.
